# Haplotypic characterization of *BRCA1 c.5266dupC*, the prevailing mutation in Brazilian hereditary breast/ovarian cancer

**DOI:** 10.1590//1678-4685-GMB-2019-0072

**Published:** 2020-05-20

**Authors:** Renan Gomes, Barbara Luisa Soares, Paula Silva Felicio, Rodrigo Michelli, Cristina B. O. Netto, Barbara Alemar, Patrícia Ashton-Prolla, Edenir Inêz Palmero, Miguel Ângelo Martins Moreira

**Affiliations:** 1 Instituto Nacional de Câncer, Programa de Genética, Rio de Janeiro, RJ, Brazil.; 2 Hospital de Cancer de Barretos, Centro de Pesquisa em Oncologia Molecular, Barretos, SP, Brazil.; 3 Hospital de Clínicas de Porto Alegre, Serviço de Genética Médica, Porto Alegre, RS, Brazil.; 4 Hospital de Clínicas de Porto Alegre, Laboratório de Medicina Genômica, Porto Alegre, RS, Brazil.; 5 Universidade Federal do Rio Grande do Sul (UFRGS), Departamento de Genética, Programa de Pós-Graduação em Genética e Biologia Molecular, Porto Alegre, RS, Brazil.; 6 Faculdade de Ciências de Saúde de Barretos Dr. Paulo Prata (FACISB), Barretos, SP, Brazil.

**Keywords:** Founder mutation, Ashkenazi Jewish, BRCA1, BRCA1 c.5266dupC, hereditary breast cancer

## Abstract

Specific pathogenic mutations associated with breast cancer development can vary between ethnical groups. One example is *BRCA1 c.5266dupC* that was first described as a founder mutation in the Ashkenazi Jewish population, but was later also found in other populations. In Brazil, this mutation corresponds to 20% of pathogenic *BRCA1* variants reported. Our objective was to investigate the haplotype component of a group of Brazilian families who inherited *c.5266dupC* in the *BRCA1* gene and to verify the ancestry contribution from European, African, and Amerindian origins. Fourteen probands carrying *c.5266dupC* and 16 relatives (carriers and non-carriers) were investigated. The same haplotype was observed segregating within all the families analyzed, revealing no recombinants in a region of 0.68 Mb. Ancestry analysis demonstrated that the European component was predominant among probands. The *BRCA1 c.5266dupC* analysis indicates that there was a founder effect in the Brazilian population.

The spectrum of pathogenic mutations found in genes related to cancer development can vary depending on the ethnic groups that are being studied. Specific pathogenic mutations associated to particular ethnic groups show a high frequency due to founder effects, or population bottleneck and consequent inbreeding. As a result, rare pathogenic mutations become more common within the population over time (Ferla *et al.*, 2007). Inbreeding contributes to linkage disequilibrium in genomic regions that are segregated together for many generations with specific alleles in *loci* placed closest to the mutation site in that specific population (Fackenthal and Olopade, 2007).

A well-known example of founder effects is that of *BRCA* pathogenic mutations in the Ashkenazi Jewish population (Rubinstein, 2004). At least 2.6% (1/40) of this population carry one of the three founder pathogenic mutations described for *BRCA1* (OMIM #113705) and *BRCA2* (OMIM #600185): *BRCA1c.66_67delAG* (p.Glu23fs), *BRCA1 c.5266dupC* (p.Gln1756fs, former named 5382insC), and *BRCA2c.5946delT* (p.Ser1982fs) (Rubinstein, 2004; Antoniou *et al.*, 2005; Ferla *et al.*, 2007; Hamel *et al.*, 2011; Tafe *et al.*, 2015). Although these pathogenic mutations were first identified in Ashkenazi Jews, *BRCA1 c.5266dupC*, was later described in other populations. It has already been identified in many countries of Central and Eastern Europe (Burcos *et al.*, 2013; Gorodetska *et al.*, 2015) and also recurrently described in the Brazilian population (Lourenço *et al.*, 2004; da Costa *et al.*, 2008; Fernandes *et al.*, 2016), representing 20% of the BRCA1 pathogenic variants reported in a recent survey (Palmero *et al.*, 2018).

The comparison of haplotypes between families sharing the same mutation allows to distinguish whether high-frequency alleles derive from a single mutational event (a founder mutation), or if they have arisen independently more than once in a population (Hamel *et al.*, 2011; Ossa and Torres, 2016). In a previous report, da Costa *et al.* (2008) showed that seven unrelated carriers of this mutation share the same haplotype of genetic marker alleles flanking *BRCA1*. However, data available in Brazil regarding the frequency of different pathogenic variants in individuals at risk of hereditary breast/ovary cancer and the availability of samples from the c.5266dupC mutation carriers limited the conclusions at that time with respect to a possible founder effect. In addition, da Costa *et al.* (2008) did not evaluate the ancestry of the carriers.

In this work, we present a haplotype analysis in an expanded set of Brazilian carriers of *BRCA1 c.5266dupC*. In addition, considering that the Brazilian population is highly admixed, with genetic backgrounds derived from Europeans, Africans and Amerindians, ancestry analysis was carried out to assess the contribution of these different backgrounds to the genetic diversity present in the carriers

Fourteen unrelated heterozygous probands harboring *BRCA1 c.5266dupC* and 26 relatives (carriers or non-carriers of eight families) were recruited for this study from collaborating research centers located in the cities of Rio de Janeiro (5 probands), Barretos (5 probands), and Porto Alegre (4 probands) between 2004 to 2016. All research procedures followed ethical guidelines and were approved by the local Ethics Committee (009/07). The five probands/families from Rio de Janeiro were also previously analyzed in da Costa *et al.* (2008).

Haplotypes were characterized based on three SNPs and four Short Tandem Repeat (STR) markers along ~581 kb of chromosome 13 encompassing the *BRCA1* locus (Figure S1). SNPs were analyzed by PCR amplification and DNA sequencing, as described by da Costa *et al.* (2008).

Four microsatellite *loci* were used for genotyping: D17S855 (intragenic marker in intron 20), D17S1325 and D17S1326 (3’ markers) and D171321 (5’ marker); primers are listed in Table S1. For all microsatellite loci, PCR amplifications were performed in final volumes of 25 μL, with 2.0 mmol/L of MgCl2, 125 μmol/L of each dNTP, 20 pmol of each primer, 1x PCR buffer, 1 U of Platinum *Taq* DNA polymerase (Invitrogen) and 50 ng of genomic DNA. Reactions were submitted to 30 cycles of 94 °C for 15 s, 60 °C for 15 s, and 72 °C for 20 s. Forward primers were labeled with carboxyfluorescein, and PCR products were analyzed in an ABI-PRISM 3730 automatic sequencer (Applied Biosystems, Foster City, CA). Scoring of allele size was achieved using the internal size standard GeneScan –500 LIZ^®^ (Applied Biosystems). Allele size was estimated using Peak Scanner^TM^ software v1.0 (Applied Biosystems).

To estimate genetic ancestry, 46 ancestry-informative markers (AIMs) were selected. These markers were used to investigate the contribution of African, European, East Asian, and Native American populations to the genetic background of the probands. AIMs were genotyped in one multiplex PCR assay followed by capillary electrophoresis, as previously described (Pereira *et al.*, 2012). *Structure* software (Pritchard *et al.*, 2000) was used to estimate the ancestral components of the samples, and the results were validated by *Admixture* software. Genetic ancestry analysis was carried out for the 14 index cases.

Of all *BRCA1 c.5266dupC* carriers, 21 (63.6%) had been diagnosed with breast and/or ovarian cancer at ages ranging from 22 and 63 years old (median = 43 years) (Table 1). The age of cancer-unaffected mutation carriers (n=12) ranged from 22 to 66 years old (median = 47). For five of the 14 probands only the personal cancer history was available, for the remainder at least one relative developed breast or ovarian cancer (Table 1).

**Table 1:** Clinical features of *BRCA1 c.5266dupC* carriers.

Carrier	Relationship	Gender	Cancer type	Tumor location	Age at diagnostic
RJ-01	Proband	F	Ovarian	Bilateral	47/47
RJ-02	Proband	F	Breast	Bilateral	36/41
RJ-02C	3^rd^ degree relative	F	Breast	Bilateral	47
RJ-02F	3^rd^ degree relative	F	Breast	Unilateral	49
RJ-02G	4^th^ degree relative	F	Healthy	-	-
RJ-03	Proband	F	Breast	Bilateral	47/47
RJ-04	Proband	F	Breast	Bilateral	33/38
RJ-05	Proband	F	Breast	Unilateral	33
RJ-05A	1^st^ degree relative	F	Healthy	-	-
SP-01	Proband	F	Breast	Bilateral	36
SP-01A	2^nd^ degree relative	F	Breast	Bilateral	46
SP-01B	3^rd^ degree relative	F	Healthy	-	-
SP-01C	5^th^ degree relative	F	Healthy	-	35
SP-02	Proband	F	Breast	Unilateral	63
SP02-A	1^st^ degree relative	F	Healthy	-	-
SP-03	Proband	F	Breast	Bilateral	37
SP-03A	1^st^ degree relative	F	Breast	Unilateral	41
SP-03B	1^st^ degree relative	M	Healthy	-	-
SP-04	Proband	F	Breast	Bilateral	31
SP-04A	1^st^ degree relative	F	Healthy	-	-
SP-05	Proband	F	Ovarian	-	49
SP-05A	1^st^ degree relative	F	Healthy	-	-
SP-05B	1^st^ degree relative	F	Breast	Unilateral	47
SP-05C	1^st^ degree relative	F	Healthy	-	-
SP-05D	3^rd^ degree relative	M	Healthy	-	-
SP-05E	3^rd^ degree relative	F	Healthy	-	-
RS-01	Proband	F	Breast	Bilateral	36/-
RS-01A	3^rd^ degree relative	F	Ovarian	Bilateral	52/54
RS-02	Proband	F	Breast	Bilateral	23/44
RS-03	Proband	F	Breast	Bilateral	-
RS-04	Proband	F	Breast	Bilateral	35/45
RS-04A	1^st^ degree relative	F	Healthy	-	-
RS-05B	3^rd^ degree relative	F	Breast	Bilateral	49/64

Fourteen probands carrying *BRCA1 c.5266dupC* and 26 relatives (carriers and non-carriers) were haplotyped. The same haplotype associated with *c.5266dupC* was segregating within all the families analyzed, revealing no recombinants in a region of 0,68 Mb (Figure S2). On the other hand, this haplotype was not found in non-carrier relatives analyzed (n=7). Ancestry analyses showed that the European component was predominant among the probands, with an average of 81.15% (Figure 1).

**Figure 1:**
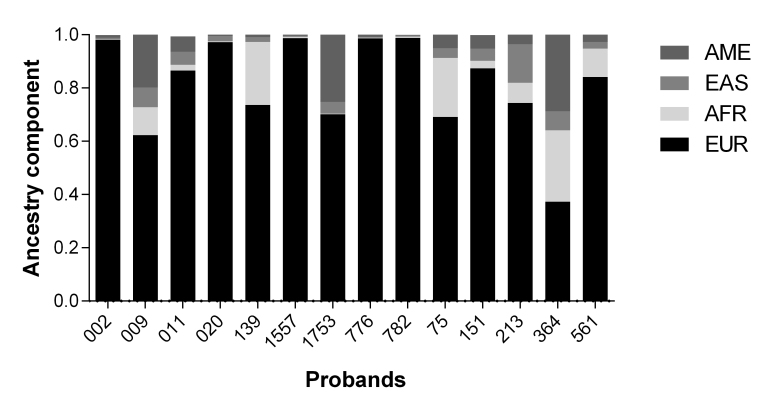
Ancestry components of the 14 probands analyzed. Ancestry analysis was performed to investigate the contribution of African (AFR), European (EUR), East Asian (EAS), and Native American (AME) populations in our probands.

The high frequency of breast cancer observed in our sample, especially bilateral cancer (n=13/21), was also reported in other studies and recently associated to its location (within the breast cancer cluster region of the gene) (Rubinstein, 2004; Hamel *et al.*, 2011; Pritchard *et al.*, 2012; Rebbeck *et al.*, 2015; Tafe *et al.*, 2015). The segregation of the same haplotype within *BRCA1 c.5266dupC* in all carrier relatives analyzed, reinforced the founder effect of this mutation in the Brazilian population.

Our data is in accordance with previous results showing a European component that exceeds 70% in the South and Southeast of Brazil (Kehdy *et al.*, 2015), and support the European origin of *BRCA1 c.5266dupC* in Brazil. The predominant European ancestry observed in the carriers studied here is in line with the proposition of Hamel *et al.* (2011) for the Scandinavian origin of this mutation, followed by its dispersion through Central Europe 400-500 years ago. As was stated before (da Costa *et al.* , (2008)), a better explanation for the presence of this mutation in the Brazilian population is the immigration from Central Europe diring the 19^th^ century encouraged by Brazilian officials (Pena *et al.*, 2011), particularly to the Southeast and South regions of Brazil (Kehdy *et al.*, 2015), where the mutation is nowadays more frequently found.

There are some limitations to the present study. Unfortunately, the sample size was small, considering that *c.5266dupC* corresponds to 20.2% of all Brazilian *BRCA1* pathogenic variants (Palmero *et al.*, 2018), although, our samples account for patients of three Brazilian states. Nonetheless, it is the largest published study revealing a single haplotype between carriers in Brazil. This unique haplotype validates the founder effect for the *c.5266dupC* insertion in Brazil, and the ancestry data reveal the contribution of Central Europe for the Brazilian genetic background. The frequency of this mutation is shown to be relevant especially among patients of the southern and utheastern Brazilian regions, where the European ancestry contribution is large. Our study also shows that *c.5266dupC* is associated with the appearance of bilateral breast tumors, which confirms what was previously observed by other authors (Ewald *et al.*, 2011). Considering a scenario of limited resources, low cost screening focused on this recurrent pathogenic variant could be offered for patients and their families of European ancestry. However, this strategy is not adequate in view of the diversity of pathogenic *BRCA1* and *BRCA2* variants found in Brazil and the admixed ethnic origin of its individuals.

## Supplementary material

The following online material is available for this article:

Table S1 - Primers sequences used for genotyping.

Figure S1 - Seven molecular markers used in haplotype analysis.

Figure S2 - Pedigree of probands’ families and haplotype analyses.
