# Supplementary material for: Haplotypic characterization of BRCA1 c.5266dupC, the prevailing mutation in Brazilian hereditary breast/ovarian cancer
Source: Genet Mol Biol. 2020 May 20;43(2):e20190072. doi: 10.1590//1678-4685-GMB-2019-0072 (PMC7250276; doi:10.1590//1678-4685-GMB-2019-0072)
Supplement: Table S1 [file 1415-4757-GMB-43-2-e20190072-s1.pdf]

Supplementary Material to "Haplotypic characterization of *BRCA1 c.5266dupC*, the prevailing mutation in Brazilian hereditary breast/ovarian cancer"

Table S1 - Primers sequences used for genotyping.

Locus	Primer sequence (5' → 3')	Product size
D17S1321	CAGAGTGAGACCTTGTCTCAAA<br>TTCTGCAAACACCTTAAACTCAG	170 pb
D17S855	GGATGGCCTTTTAGAAAGTGG<br>ACACAGACTTGTCCTACTGCC	150 pb
D17S1326	CAGCTGATATTTCACAGGACT<br>AGAGCAAAACTCCATCTCAAACA	90 pb
D17S1325	AAAGGTGGCAATTCACAGTTG<br>GTGATAAAACTCAGTGGTACTC	155 pb
rs799905	TAGCCCCTTGGTTTCCGTG<br>TCACAACGCCTTACGCCTC	315 pb
rs16941	CCAGTACAGTGAGCACAATTA<br>GTGTTGGAAGCAGGGAAGCTCTTC	626 pb
rs1799966	AATTCTTAACAGAGACCAGAAC<br>AAAACTCTTTCCAGAATGTTGT	450 pb
5266dupC	ATATGACGTGTCTGCTCCAC<br>GGGAATCCAAATTACACAGC	410 pb
