# Supplementary material for: Haplotypic characterization of BRCA1 c.5266dupC, the prevailing mutation in Brazilian hereditary breast/ovarian cancer
Source: Genet Mol Biol. 2020 May 20;43(2):e20190072. doi: 10.1590//1678-4685-GMB-2019-0072 (PMC7250276; doi:10.1590//1678-4685-GMB-2019-0072)
Supplement: Figure S1 [file 1415-4757-GMB-43-2-e20190072-s2.pdf]

Supplementary Material to "Haplotypic characterization of *BRCA1 c.5266dupC*, the prevailing mutation in Brazilian hereditary breast/ovarian cancer"

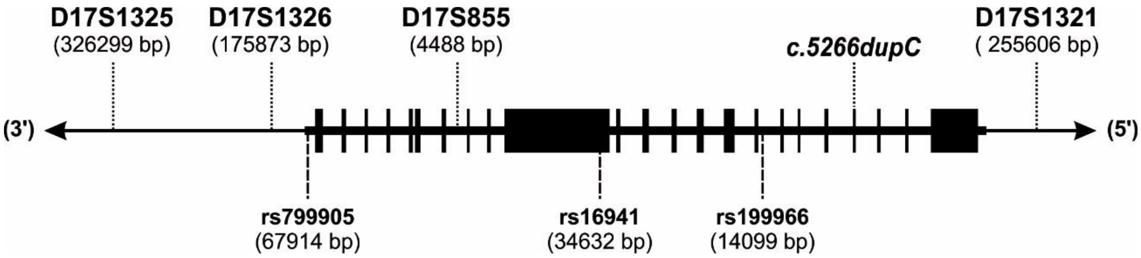

Figure S1 - Seven molecular markers used in haplotype analysis. The values above each marker represent the distance, in kilobases (Kb), between the markers and *c.5266dupC* insertion.
