# Supplementary material for: Haplotypic characterization of BRCA1 c.5266dupC, the prevailing mutation in Brazilian hereditary breast/ovarian cancer
Source: Genet Mol Biol. 2020 May 20;43(2):e20190072. doi: 10.1590//1678-4685-GMB-2019-0072 (PMC7250276; doi:10.1590//1678-4685-GMB-2019-0072)
Supplement: Figure S2 [file 1415-4757-GMB-43-2-e20190072-s3.pdf]

Supplementary Material to "Haplotypic characterization of *BRCA1 c.5266dupC*, the prevailing mutation in Brazilian hereditary breast/ovarian cancer"

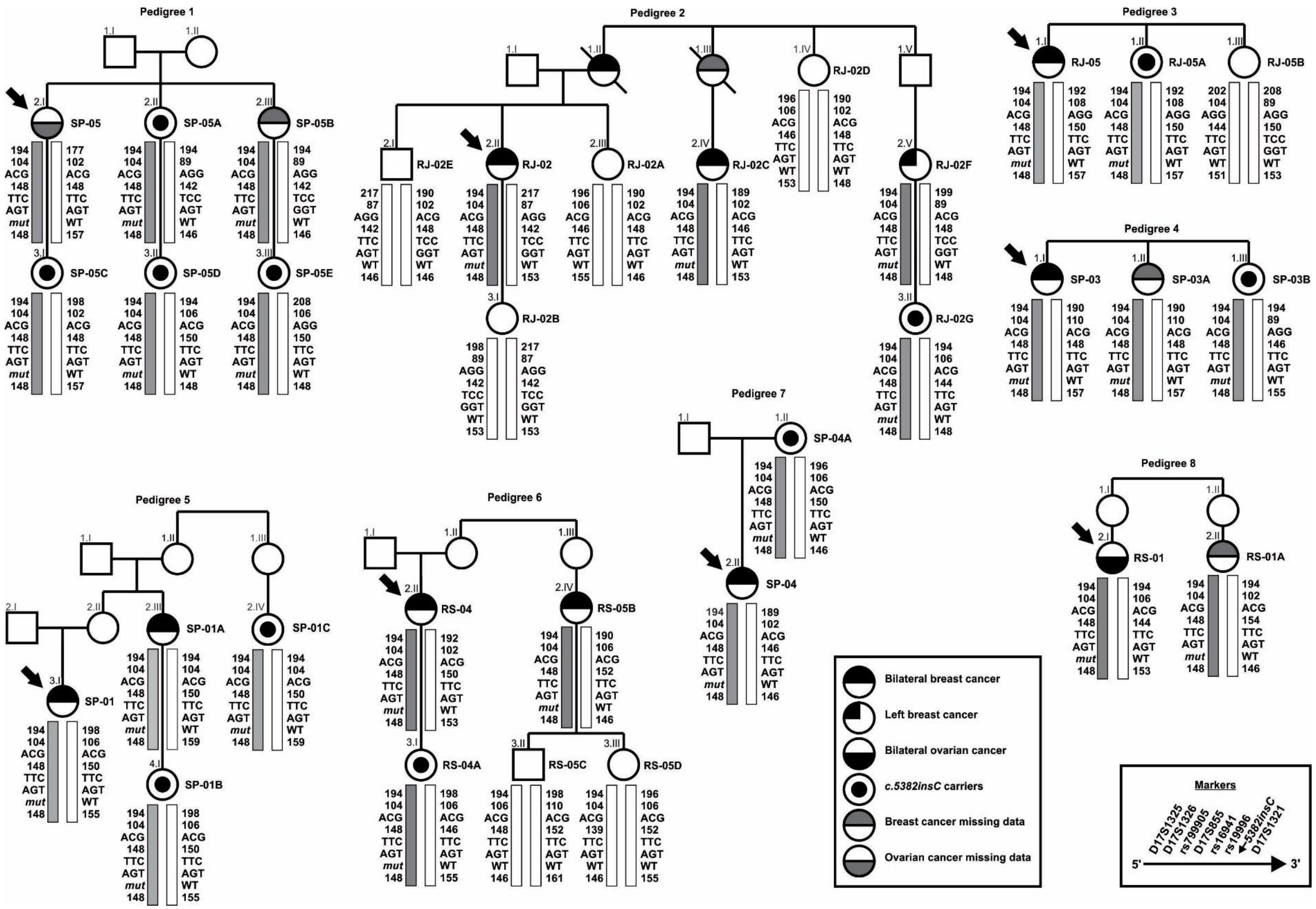

Figure S2 - Pedigree of probands' families and haplotype analyses. The haplotype linked to the mutation is gray painted. The phenotype features are given in the bottom right box and the markers used are given in the center box.
